# Artificial sweeteners and their implications in diabetes: a review

**DOI:** 10.3389/fnut.2024.1411560

**Published:** 2024-06-25

**Authors:** Matcha Angelin, Janardhanan Kumar, Leela Kakithakara Vajravelu, Abhishek Satheesan, Venkata Chaithanya, Ria Murugesan

**Affiliations:** ^1^Department of Microbiology, SRM Medical College Hospital and Research Centre, SRM IST, Kattankulathur, Tamil Nadu, India; ^2^Department of General Medicine, SRM Medical College Hospital and Research Centre, SRM IST, Kattankulathur, Tamil Nadu, India

**Keywords:** sweeteners, diabetes mellitus, artificial sweeteners, glucose, gut microbiota

## Abstract

Diabetes is a significant global health concern, highlighting the critical role of dietary strategies in its management and prevention. Artificial sweeteners (ASs), due to their capacity to provide sweetness without contributing to caloric intake, have emerged as a potential tool in diabetes management. This review thoroughly examines the nuanced relationship between artificial sweeteners and diabetes, addressing their benefits and potential risks. ASs have been shown to aid in weight management, a key factor in reducing diabetes risk, and do not impact immediate blood glucose levels, offering improved glucose control for individuals with diabetes. Beyond these benefits, however, artificial sweeteners may interact complexly with gut microbiota, potentially altering its composition and affecting metabolic health. This interaction introduces concerns regarding insulin sensitivity and the risk of insulin resistance, with studies reporting conflicting findings. This comprehensive review highlights the importance of a nuanced approach to understanding the implications of artificial sweeteners in diabetes management. Given the mixed evidence on their health effects, there is a clear need for further research to fully elucidate the role of artificial sweeteners in metabolic health and their suitability as part of dietary interventions for diabetes.

## 1 Introduction

Globally, in the year 2021, there were approximately 536.6 million adults between the ages of 20 and 79 who have diabetes, representing about 10.5% of the adult population. By 2045, this figure is projected to reach 783.2 million, making up about 12.2% of the population (1). Diabetes Mellitus (DM) is a chronic health condition characterized by high levels of sugar (glucose) in the blood, often due to the body's inability to effectively regulate blood sugar levels. This glucose metabolic disorder can lead to various complications and requires lifelong management, typically through medication, diet, and lifestyle changes (2).

DM is made of various subtypes. Although the primary types are Type 1 DM (T1DM) and Type 2 DM (T2DM), others include gestational diabetes, steroid-induced diabetes, maturity-onset diabetes of the young (MODY), and neonatal diabetes. Every of these types (T1DM and T2DM) have a distinct pathophysiological characteristics, clinical presentations, and treatment approaches, but, both types have the potential to lead to elevated blood sugar levels (3). The primary subtypes are Type 1 diabetes mellitus (T1DM) and Type 2 diabetes mellitus (T2DM), which typically arise from impaired insulin secretion (T1DM) and/or insulin function (T2DM). T1DM are mostly found in children or adolescents, while T2DM is often associated with middle-aged and older adults who have experienced prolonged hyperglycemia due to unhealthy lifestyle and dietary choices (3).

T2DM is recognized as a serious public health concern with a considerable impact on human life and health expenditures. In 2017, approximately 6.3% of adults worldwide were diagnosed with T2DM, and this number is projected to increase to 7.4% by 2040 (4). T2DM is often accompanied by comorbidities such as high blood pressure, dyslipidemia, and an increased risk of cardiovascular events. It is widely recognized that diet plays a crucial role in preventing T2DM. Artificial sweetener (ASs) are often used as a characteristic feature of ultra-processed foods (UPFs), which have been found to be linked with an increased risk of developing T2DM (5). ASs, also known as non-nutritive sweeteners (NNS) or high-intensity sweeteners, are substances used to add sweetness to food and drinks without the addition of extra calories from sugar or high fructose corn syrup (HFCS). These sweeteners serve as substitutes for sugar and HFCS, providing little to no calories or nutritional value. Additionally, they are significantly sweeter than traditional sugar, often hundreds to thousands of times sweeter in comparison (5, 6). The increased usage of ASs is often influenced by their potential for assisting in weight management and controlling blood glucose levels (6). They are extensively utilized in a wide range of beverages and food products, including diet soft drinks, yogurts, desserts, and chewing gum. Many food manufacturers opt to use a combination of NNS or a mixture of sugar and NNS to enhance the flavor and overall palatability of products containing these sweeteners (7). Substituting ASs for sugars holds promise in reducing sugar and energy intake since these sweeteners provide a sweeter taste without adding calories (8). This review highlights the use of ASs and its implications in diabetes.

## 2 Methodology

This review was carried out using an electronic literature search by reviewing publications on Google, PubMed, Google Scholar, and MedLine Plus. We selected articles that contained keywords such as “artificial sweeteners,” “diabetes,” “obesity,” “non-caloric artificial sweeteners,” “sugar substitutes,” and “non-nutritive sweeteners,” among others. After the initial selection, we examined and included articles that were relevant to the topic into this paper.

### 2.1 Global impact and epidemiology of diabetes mellitus

Due to swift economic growth, improved living standards, changes in dietary habits, shifts in lifestyle, and the aging population, DM has emerged as a significant global public health issue (9–11). It is now estimated to be the third most challenging health issue after cancer and cardiovascular diseases (12).

A recent investigation revealed a rapid increase in the occurrence of DM, with a notable surge in developing nations, especially in Asia (13). Furthermore, Asia, being the most densely populated region globally, is home to over 60% of the diabetic population worldwide. Projections suggest a significant rise in the number of individuals living with diabetes in every Asian country in the coming decades (13).

Briefly, the etiology of DM has it that, the pancreas's islets of Langerhans have two primary types of endocrine cells: beta cells, responsible for producing insulin, and alpha cells, which secrete glucagon. These cells adjust their hormone secretion levels in response to glucose levels in the body. Maintaining a delicate balance between insulin and glucagon is crucial; otherwise, blood glucose levels become imbalanced. In the case of DM, insulin is either absent or functions inadequately (insulin resistance), resulting in elevated blood sugar levels. T1DM is characterized by the destruction of beta cells in the pancreas, typically due to an autoimmune process. This leads to the complete loss of beta cells and, consequently, very low or no insulin production. T2DM has a more gradual onset, where an imbalance between insulin production and insulin sensitivity causes a functional deficiency in insulin. Insulin resistance, a common factor in T2DM, often develops due to factors such as obesity and aging. Genetic makeup of individual also plays a crucial role in the risk of both types of diabetes. Precision medicine and nutrigenomics offer tailored approaches to diabetes management by considering individual genetic variations (14). These approaches aim to optimize treatment and dietary recommendations based on genetic profiles, enhancing the effectiveness of interventions and potentially improving patient outcomes (15). Ongoing research into the human genome has identified various loci associated with an increased risk of DM. Polymorphisms, or genetic variations, have been shown to influence the risk of T1DM, including those in the major histocompatibility complex (MHC) and human leukocyte antigen (HLA) (16).

T2DM has a more intricate interaction between genetics and lifestyle. There is major evidence indicating that T2DM has a stronger genetic predisposition when compared to T1DM. A significant proportion of individuals diagnosed with this condition have at least one parent with T2DM (17). On a global scale, DM affects 1 in every 11 adults, with 90% of these cases being attributed to T2DM (18). The onset of T1DM gradually rises from birth, reaching its highest prevalence between the ages of 4–6 years and then again between 10 and 14 years (18). T2DM typically begins at a later stage of life; however, the rise of obesity among teenagers has resulted in a higher incidence of T2DM among younger individuals. In the United States, T2DM affects approximately 9% of the entire population but is prevalent in around 25% of those aged 65 and older.

T2DM vary among different ethnic groups, with a prevalence that ranges from 2 to 6 times higher in populations such as Black people, Native Americans, North American natives (Pima), and Hispanic Americans compared to Caucasians in the United States (19, 20). While ethnicity is a significant factor in T2DM risk, environmental influences also play a substantial role in the development of the disease. For instance, North American natives (Pima) in Mexico have a lower likelihood of developing T2DM compared to their counterparts in the United States, with prevalence rates of 6.9% and 38%, respectively (21).

Although T1DM and T2DM can have similar presentations, they show differences in clinical history and examination. Patients with T2DM typically exhibit signs of insulin resistance and are often overweight or obese. Additionally, they may display acanthosis nigricans, characterized by hyperpigmented and velvety patches on the skin located in areas like the neck, axillary regions, or inguinal folds.

Diabetes can impact various organ systems in the body, including the nervous system, kidneys, and eyes, potentially leading to severe complications over time (22, 23). Recent meta-analyses have confirmed that individuals with diabetes mellitus face roughly twice the risk of developing large vessel diseases like coronary heart disease (CHD) and stroke (24) as well as nonvascular mortality (25). Consequently, there has been widespread promotion of efforts to control the increasing prevalence of DM in order to reduce the risk of these large-vessel diseases. Both pharmaceutical treatments for DM and lifestyle modifications have demonstrated effectiveness in reducing the incidence of this condition (26).

Data from European countries have revealed that healthcare costs for patients with diabetes mellitus are significantly higher compared to those without the disease (27–29).

## 3 Types of sweeteners and their biochemical effects

**Figure 1:**
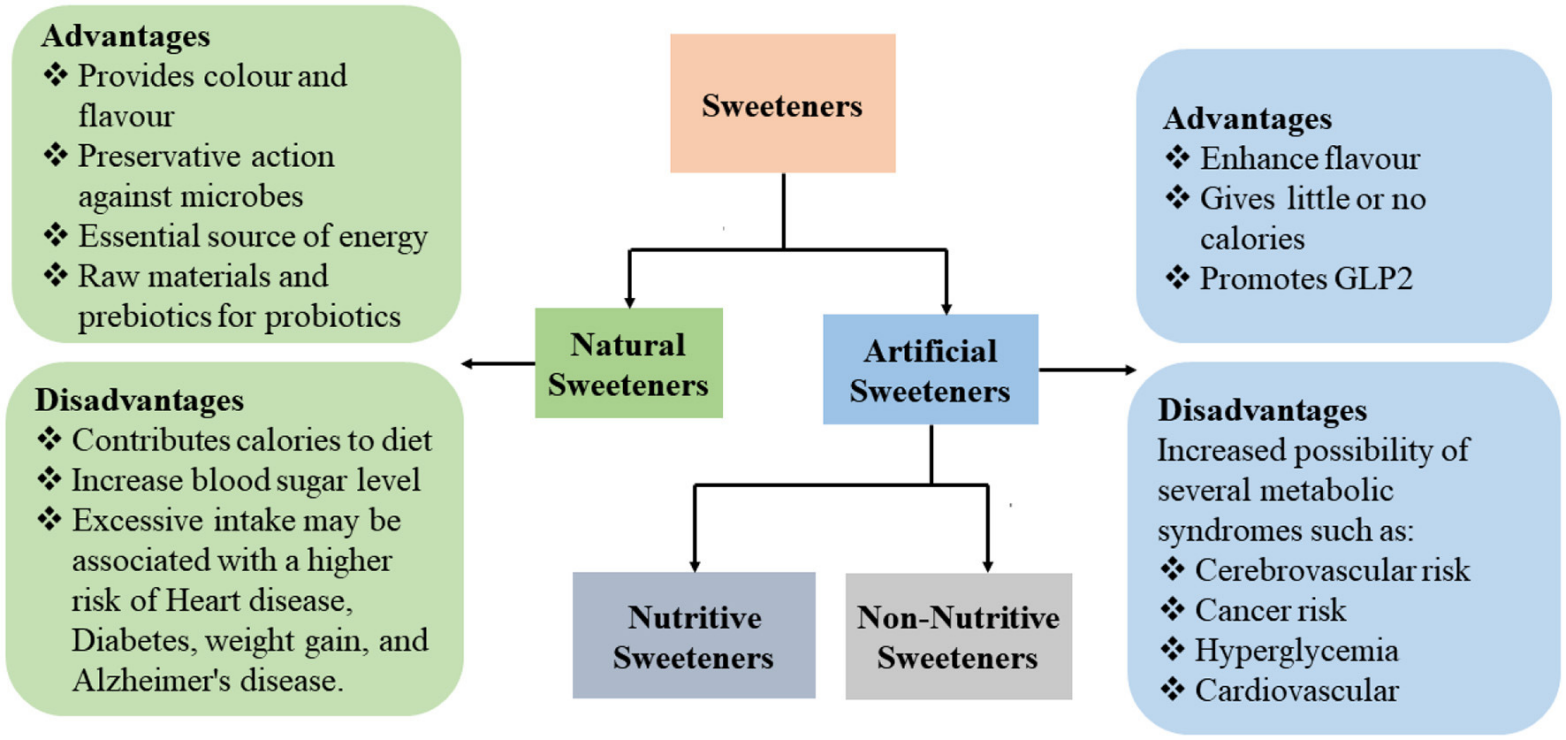
Types of sweeteners, advantages and disadvantages of natural and ASs.

Sweeteners are classified as natural sweeteners and ASs (30), with each having its own advantages and disadvantages (30) (Figure 1).

### 3.1 Natural sweeteners

Natural sweeteners can be found or are created by nature without the use of chemicals or sophisticated machinery. Only naturally occurring sugars and carbohydrates found in live plants such as vegetables, trees, seeds, nuts, and roots are healthy to consume, along with wild, non-hybridized, seeded fruits. Natural sweeteners consist of the following: Xylitol, coconut sugar, date sugar, coconut nectar, honey, stevia, molasses, and maple syrup (8). In the 18th century, the fast expansion of sucrose extraction from sugar beet and sugar cane eclipsed honey, which had previously been the main source of sweetness in human diets. The most popular sweetener today is sucrose, also referred to as table sugar and now offered in many refined forms. Consumption of sucrose has increased recently, reaching 174 million metric tons in 2018 and 2019 (31). Unfortunately, excessive sugar consumption has expanded and is now a significant problem with negative health effects. The research demonstrates a strong association between consuming too much sugar and an elevated risk of cardiovascular disease, type 2 diabetes, obesity, dental caries, and other non-communicable diseases (31).

### 3.2 Artificial sweeteners: overview

Although sugar substitutes called ASs have been in existence since the 1880s, there has been a substantial rise in the consumption of ASs over the past two decades. This increase has garnered more attention as these sweeteners are considered tools for dietary assessment, aiming to address the obesity epidemic by providing a sweet taste without the added calories (32). Taste plays a crucial role in how humans perceive the quality of food, significantly contributing to their overall satisfaction and enjoyment. In light of this, the development of sweeteners as food additives that replicate the natural sweetness of sugar holds promise (33).

ASs are further categorized as nutritive and non-nutritive sweeteners based on whether or not they include calories. The monosaccharide polyols (such as xylitol, mannitol, and sorbitol) and disaccharide polyols (such as lactitol and maltitol) are examples of natural nutritive sweeteners. The NNS, known as ASs, include substances from different chemical classes that are 30–13,000 times sweeter than sucrose.

These NNS with low calorie alternatives that provide little or no energy has led to them becoming a common substance of the Western diet (34). Cross-sectional studies have revealed that 25% of children and 41% of adults regularly include low-calorie sweeteners in their diets. The consumption of NNS is notably higher among females, individuals who are obese, non-Hispanic white individuals, and those with higher incomes (35, 36). Interestingly, one study conducted a comprehensive assessment of 24 NNS, examining their presence in the environment across 38 locations worldwide, including Europe, the United Kingdom, Canada, the United States, and Asia. The study's overall findings indicated that NNS are detectable in various environmental sources, including surface water, tap water, groundwater, seawater, lakes, and even the atmosphere (37).

ASs have a variety of qualities, including sweetness intensity, sweetness duration, coating of the teeth, and aftertaste effects, they are also metabolized differently (38). To some extent, ASs protect against obesity and insulin resistance by counteracting Lipopolysaccharide (LPS)—induced inflammation and consequent impairment of insulin signaling (30). They have a different effect on body weight and glucose homeostasis than natural sugars because of underlying physiological mechanisms such as the gut microbiota, reward system, adipogenesis, insulin secretory capability, intestinal glucose absorption, and insulin resistance (30).

Many people turn to NNS as a means to reduce their daily calorie intake, manage weight, and maintain a healthy diet. Despite their widespread use, there is scientific evidence to support the safety of NNS consumption. However, recent research has indicated that the use of NNS may disrupt the balance of gut microbiota and lead to impaired glucose tolerance in otherwise healthy individuals, potentially contributing to the development of T2DM (39).

### 3.3 Types and chemical composition of artificial sweeteners

**Figure 2:**
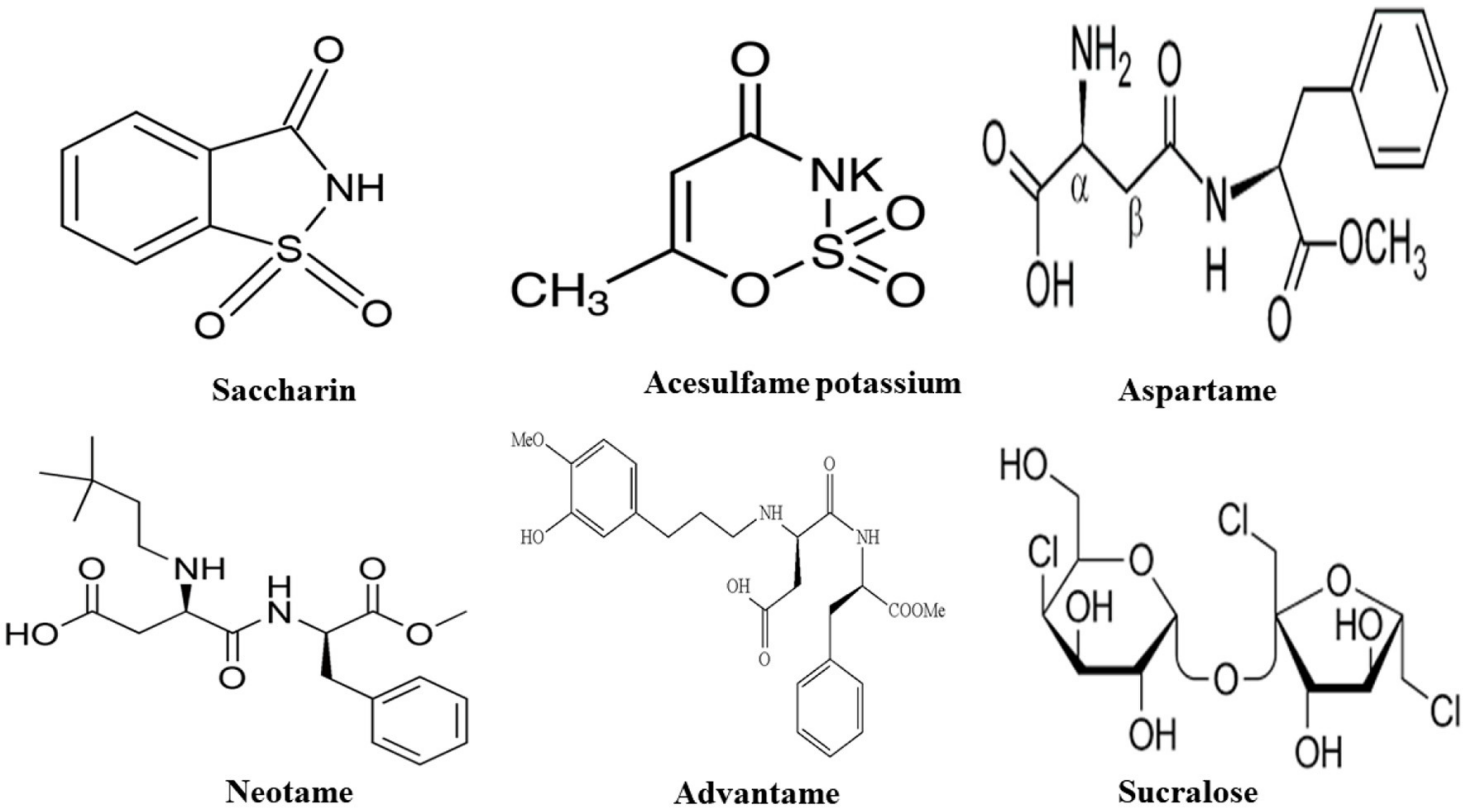
Types and chemical structure of ASs.

Presently, the Food and Drug Administration (FDA) has granted approval for the usage of high-intensity sweeteners: acesulfame-potassium (Ace-K), aspartame, neotame, saccharin, sucralose, and purified form of stevia such as rebaudioside A, which is recognized as ASs (39). The European Union has a broader range of approved ASs, including cyclamate, for example. While stevia has been utilized as a sweetener in certain countries like Japan for many years, it received recent approval as a food additive from both the European Food Safety Authority (EFSA) and the U.S. FDA (40). Below is the list of ASs discussed in this paper (Figure 2, Table 1).

**Table 1:** Outlined properties of artificial sweeteners.

**Artificial sweeteners**	**Brand name**	**No. of calorie (kcal)**	**Relative sweetness to sucrose**	**Acceptable daily intake (mg/kg bw/d)**	**Reaction to heat**	**Bitter after taste**	**Application in food industry**
Saccharin (39, 41)	Sweet and Low^®^, Sweet Twin^®^, Sweet'N Low^®^, Necta Sweet^®^	0	300	5	Stable	Yes	Beverages, bases, and mixes for many food products, table sugar substitute
Acesulfame potassium (39, 41)	Sunett^®^, Sweet One^®^	0	200	15	Stable	Yes	Beverages, candy, frozen desserts, baked goods. Heat stable so it can be used in baking
Aspartame (39, 41)	Nutrasweet^®^, Equal^®^, Sugar Twin^®^	4	180–200	50	Not stable	No	Soft drinks, chewing gum, pudding, cereals, instant coffee. Also distributed as a “General Purpose Sweetener”
Neotame (39, 41)	Newtame^®^	0	7,000–13,000	18	Stable	No	Beverages, candy gum
Advantame (39, 41)	N/A	0	20,000	32.8	Stable	No	Baked goods, beverages, frozen desserts, frosting, chewing gum, candy, pudding, jelly and jam, gelatin
Sucralose (39, 41)	Splenda^®^	0	600	5	Stable	No	Milk, beverages, dairy products, chewing gum and ice cream.

Kcal, kilo calorie.

#### 3.3.1 Saccharin

Saccharin with a chemical formula (1,1-dioxo-1,2-benzothiazol-3-one) is an artificial sweetener that has gained widespread acceptance as a sugar alternative. It possesses a sweetness level that ranges from three hundred to five hundred times greater than sucrose and is heat stable. Serving as the most important yet commonly used sweetener, particularly for individuals with diabetes, it passes through the human digestive system without undergoing digestion (41–43). However, despite its widespread use as a sugar substitute, it is essential to consume saccharin in accordance with FDA recommended daily limit (38). Moreover, consumers should take note of both the potential advantages and risks associated with saccharin, as several studies have linked it to bladder cancer and have raised concerns about its potential involvement in other types of cancers (42).

#### 3.3.2 Acesulfame potassium (ACE-k)

Acesulfame potassium is an artificial heat stable sweetener with a sweetness level approximately two hundred times greater than sucrose which at higher concentrations may exhibit a bitter aftertaste, which is often overcome by blending it with sucralose or aspartame. This potassium salt of 6-methyl-1,2,3-oxathiazine-4(3H)-one 2,2-dioxide can be found in a wide range of products including tabletop sweeteners, carbonated beverages, frozen desserts, candies, chewing gum, dairy products, syrups, and sauces. Importantly, ACE-K undergoes no metabolic processes within the body and is eliminated through the kidneys (33, 38, 41, 44).

#### 3.3.3 Aspartame

Aspartame is a colorless and odorless powder made of methanol and two amino acids, namely aspartate and tryptophan. It boasts a sweetness level approximately two hundred times greater than sucrose. Commonly found in tabletop sweeteners, chewing gums, instant coffee, puddings, and soft drinks, aspartame is a popular choice for reducing sugar intake (33, 38, 44). Although it is a low-calorie sweetener with no impact on glycemic control, it is recommended that diabetic patients limit their consumption. Studies have indicated that aspartame can have both positive and negative effects on the lifestyle and metabolism of individuals with diabetes who rely on it (45). When compared to ACE-K (acesulfame potassium) and saccharin, aspartame does not leave a bitter aftertaste. However, it is sensitive to heat and breaks down into its constituent amino acids. Additionally, aspartame undergoes metabolism in the body, resulting in the production of methanol, aspartic acid, and tryptophan (38). Consequently, when metabolized, aspartame yields 4 kcal of energy per gram, but its caloric contribution is insignificant due to the little amount required to achieve a sweet taste (41).

#### 3.3.4 Neotame

Neotame, an aspartame analog (N-[N-(3,3-dimethylbutyl)-l-aspartyl]-l-phenylalanine 1-methyl ester), is seven thousand times sweeter than sucrose and has received FDA approval as a versatile sweetener in 2002 (38). However, it is not sold directly to consumers and is exclusively employed in food production (38). Neotame is metabolized by esterase, leading to the formation of de-esterified neotame and methanol. These byproducts are subsequently excreted through urine and feces within a span of 72 h (41, 46).

#### 3.3.5 Advantame

Advantame is chemically classified as a secondary amine of aspartame and 3-(3-hydroxy-4-methoxyphenyl) propanal (HMPA). Studies showed that 89% of the ingested advantame is eliminated through feces, while 6.2% is excreted in urine (41). Advantame also serves as a flavor enhancer for various categories such as dairy, fruit, citrus, and mint and finds its application in a range of products, including milk items, frozen dairy, nonalcoholic beverages, and chewing gums (41).

#### 3.3.6 Sucralose

Sucralose, chemically known as 1,6-dichloro-1,6-dideoxy-β-D-fructofuranosyl-4-chloro-4-deoxy-β-D-galactopyranoside is derived from sucrose, and categorized as a high-intensity sweetener (47). Numerous studies have examined the safety of sucralose, and their findings established the safety of this sweetener for human consumption (47). Sucralose is the most commonly used artificial sweetener synthesized through the chlorination of sucrose, resulting in a trichlorinated derivative of sucrose. Sucralose is approximately 600 times sweeter than sucrose and used in various products, including tabletop sweeteners, baked goods, frozen desserts, fruit juices, chewing gum, and dairy items (Table 1). Sucralose is a non-caloric sweetener that is soluble in water and stable under heat. It is not metabolized by the body, with the majority of sucralose being excreted through feces and a small percentage through urine (41).

## 4 Health outcome and NNS

Consumption of NNS can influence energy balance as well as metabolic functions through various mechanisms such as the peripheral and central systems, implying that NNS are not inert substances (48). Nevertheless, the specific mechanisms and detailed effects of NNS consumption on host metabolism and energy regulation remain to be fully understood. Although NNS consumption has been linked to several risk factors associated with metabolic syndrome (49), they are considered an option for people aiming at improving their health. Metabolic syndrome is group of factors that are known to increase the risk of developing certain disorders such as the cardiovascular disease (CVD) and T2DM. When assessed through measurements and laboratory tests, metabolic syndrome can additionally contribute to conditions such as hypertension, proinflammatory state, impaired glucose tolerance, atherogenic dyslipidemia, kidney disease and prothrombotic state (50, 51).

### 4.1 NNS and their impact on glucose dynamics

ASs are known to be a major compound in numerous products and are regularly consumed by a significant portion of the population. The consumption of dietary sugars has been linked to a range of health issue such as excess weight, CVDs, and T2DM (52). As an alternative, ASs are used as alternatives to added sugars (52). Nevertheless, an increasing body of experimental studies suggests that these sweeteners may not be as safe as previously assumed. Recently, the World Health Organization (WHO) conducted a systematic review and meta-analysis examining the relationships between ASs and health outcomes (53). Their analysis incorporated data from randomized controlled trials (RCTs), prospective studies, and case-control studies. The WHO's findings indicated potential associations between ASs and conditions such as obesity, CVD, and mortality, including a positive links with T2DM. However, the level of confidence in these associations was considered to be low (53). Other research findings suggest that the majority of sweeteners do not reveal any positive effects on DM although they have the potential to elevate the risk of developing the condition (53). Additionally, there are concerns regarding an increased risk of cancer (54, 55) and kidney disease (51) associated with sweetener consumption. ASs are often found in Ultra Processed Foods, a category of food products that has been shown to have associations with the development of T2DM (56). In their research aimed at investigating the link between ASs and the Risk of T2DM within the Prospective NutriNet-Santé Cohort, Debras and colleagues in 2022 (5) arrived at the conclusion that the consumption of ASs was associated with an elevated risk of T2DM. Specifically, they noted positive associations of T2DM with various types of sweeteners, including total sweeteners, aspartame, ACE-K, and sucralose. Interestingly, these findings differed from a study conducted within the European Prospective Investigation into Cancer and Nutrition (EPIC) cohort which suggested that an increase in daily consumption of artificially sweetened beverages (ASBs) by one can was linked to a higher risk of T2DM; however, when adjusted for body mass index (BMI), this association lost statistical significance. Likewise, in other cohorts such as EPIC Norfolk, Health Professionals Follow-Up Study (HPFS), and Black Women's Health Study (BWHS), no significant associations were found between ASBs and T2D after accounting for BMI (57).

**Figure 3:**
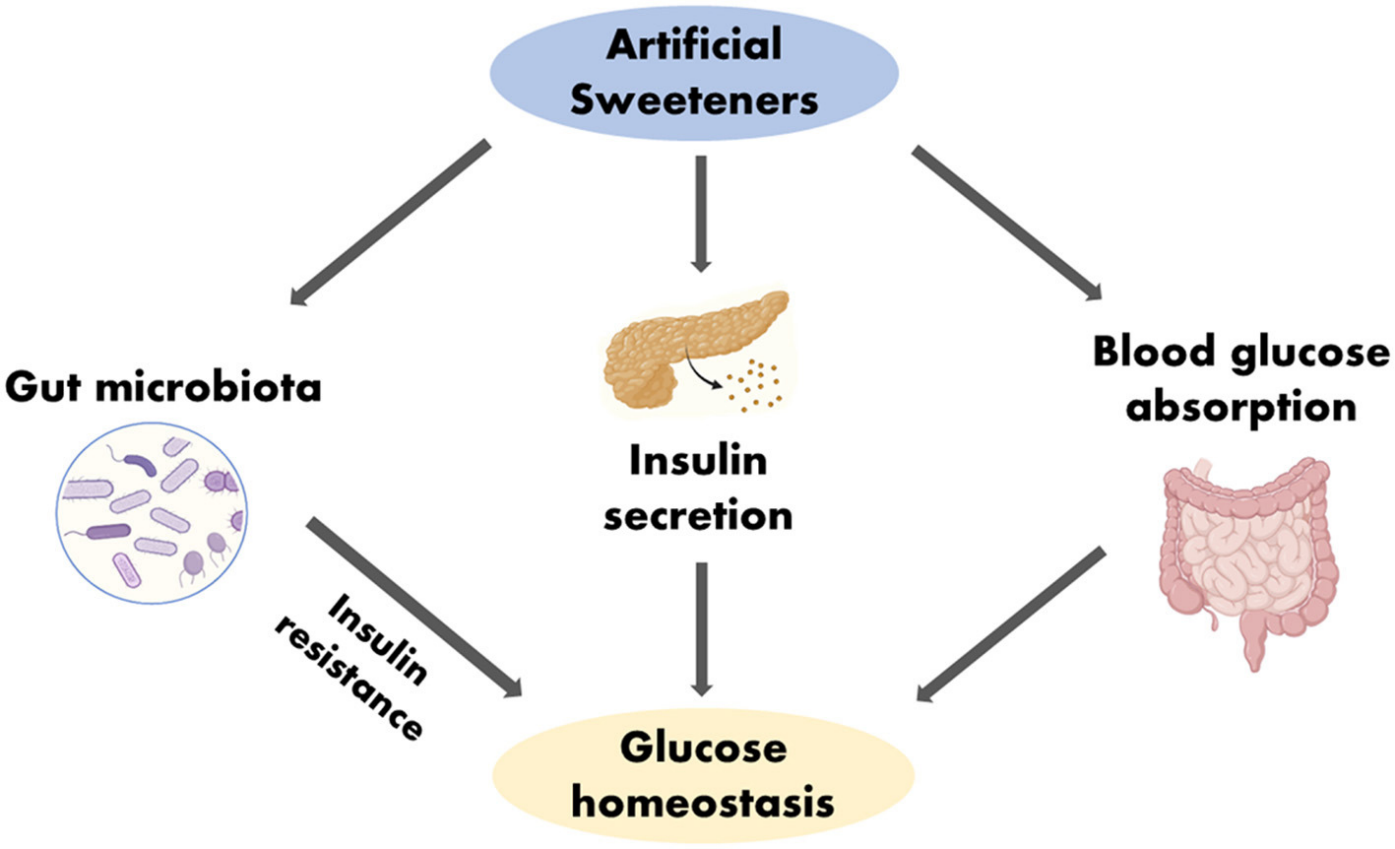
Impact of ASs in glucose homeostasis. Created with BioRender.com.

To further determine if ASs can in anyway influence the development of T2DM, certain factors such as blood glucose metabolism, insulin resistance, body weight and gut microbiota that make-up the glucose dynamics of humans needs to be considered (30, 41) (Figure 3).

#### 4.1.1 Artificial sweeteners and blood glucose

ASs has the potential to affect blood sugar levels as they reduce the absorption of glucose when they replace natural sugar (58). However, this reduction does not lead to an automatic improvement in the glucose homeostasis of the body, but instead results in alteration in the glucose homeostasis due to the changes in the intestinal glucose transport and absorption brought about by the ingestion of ASs (Figure 3). Nevertheless, the outcomes of systematic reviews and meta-analyses investigating the link between ASs consumption and glucose regulation or the risk of developing T2DM have produced contradictory results. According to Daher et al. (6) the majority of these systematic reviews and meta-analyses, which drew from randomized controlled trials (RCTs) or prospective cohort studies involving healthy individuals, did not yield definitive evidence supporting the idea that ASs heighten the risk of T2DM. Furthermore, intervention studies conducted in both healthy individuals and patients with diabetes did not identify any significant effects of ASs on factors related to glucose regulation, such as glucose and insulin levels (6). Conversely, systematic reviews and meta-analyses that relied on prospective cohort studies involving healthy individuals revealed a positive association between ASs consumption and the occurrence of T2DM, even after accounting for factors such as body adiposity (although this association was somewhat weakened after adjusting for body mass index or BMI (6). The WHO identified positive associations between ASs and elevated fasting blood glucose levels in three separate cohort studies (53). Elevated fasting blood glucose is a component of metabolic syndrome and is considered a pre-diabetic condition that can ultimately lead to T2DM (53). Other studies have shown that the consumption of ASs has no effect on glucose homeostasis in healthy adults (59). Specific types of ASs, such as aspartame and steviol glycoside, seem to have no effect on glucose homeostasis (30). Replacing natural sugars with ASs can lead to reduced caloric intake (30). NNS consumption in both mice and humans enhances the risk of glucose intolerance and that these adverse metabolic effects are mediated by modulation of the composition and function of the microbiota (60).

#### 4.1.2 Effect of NNS on insulin resistance

The consumption of nutrients involves a wide range of sensory signals that allow the human body to prepare for the digestion and utilization of these substances. Even before ingestion, exposure to sweet-tasting sugars triggers physiological responses aimed at regulating blood glucose levels, such as the release of insulin or incretin hormones. However, ASs do not elicit the same preparatory responses in the gastrointestinal (GI) tract for the digestion and utilization of nutrients as natural sugars do (30, 61).

ASs, now often used as alternatives to conventional sugars, may surprisingly influence blood sugar levels. In research conducted by Mathur et al. (62) it was observed that T2DM patients from Group A, who consumed ASs, exhibited greater insulin resistance compared to individuals in Group B, who didn't intake these sweeteners. Mathur et al., conclusion was drawn from HOMA-IR assessments. In a randomized crossover study involving healthy individuals, Smeets and colleagues (2005) (61) demonstrated that tasting aspartame did not lead to a cephalic insulin response, whereas tasting glucose resulted in an early increase in insulin concentration. Similarly, another randomized crossover study in healthy individuals reported no cephalic response when tasting sucralose (63). Additionally, while natural sugars have the ability to stimulate the secretion of incretin hormones, which in turn stimulate beta cells to release insulin, ASs do not directly induce the secretion of incretins, as this response appears to be dependent on the presence of nutrients (30, 64, 65).

#### 4.1.3 Molecular effects of artificial sweeteners on glucose and insulin signaling

In his scholarly review, Katsumi Iizuka elucidates the sophisticated interplay between ASs and metabolic health. He delineates how compounds such as sucralose, acesulfame K, aspartame, and saccharin transcend their roles as mere substitutes for sugar, influencing glucose assimilation and modulating the secretion of insulin and incretins. This nuanced interaction refutes the simplistic notion of ASs as inert substances, underscoring their profound implications on metabolic pathways via modulation of gut microbiota and endocrine responses (41). Pang et al. emphasize the intricate relationship between ASs, the regulation of body weight, and the maintenance of glucose levels, pointing out the myriad ways these substances impact metabolism. Despite being widely used as low-calorie sugar alternatives, ASs lead to diverse effects on metabolic health, attributed to the unique ways in which they are processed by the body and their distinct impacts on biological functions, including the composition of gut bacteria, the release of insulin, and the uptake of glucose. This variation highlights the critical need for more in-depth studies to decode the precise molecular actions through which various ASs affect the pathways of glucose and insulin signaling, ultimately influencing health outcomes related to metabolism (30). In our analysis, we delve into the complex ways in which NNS influence metabolic functions, leveraging the research findings of Liauchonak et al. Their study uncovers the potential for NNS consumption to disrupt the balance of gut microbiota and affect the activity of miRNAs, leading to changes in the gene expression that governs glucose metabolism and the signaling of insulin. Furthermore, Liauchonak et al. explore the pivotal interactions between IR and GPCRs, indicating that NNS may interfere with the regulation of glucose levels and sensitivity to insulin. Through this comprehensive review, informed by the insights from Liauchonak et al., we aim to shed light on the intricate biochemical interactions influenced by NNS, supporting the theory that ASs could play a role in the onset of metabolic syndrome and T2DM (39).

#### 4.1.4 Effect of NNS on body weight

It has been proposed that NNS could be a useful strategy for controlling weight (66). There is widespread agreement that excessive caloric sugar consumption, primarily through the consumption of sugar-sweetened beverages, leads to increased energy intake and poor food quality, which is linked to weight gain and/or Type 2 diabetes mellitus (T2DM). Stamataki et al. reported that the effects of daily Stevia consumption for 3 months in doses similar to real-life consumption on body weight in healthy adults with a normal body mass index (BMI) did not significantly alter their body weight from baseline, and also did not demonstrate the weight gain that occurred in the control group (67) Some of the positive results in short-term RCTs, such as weight and BMI reduction, appeared to be more evident among stevia and aspartame users (68).

#### 4.1.5 Effect of NNS on gut microbiota

The human intestinal tract houses a large assembly of over 100 trillion microbial cells. This ecosystem serves critical functions in regulating metabolism. By engaging in mutually beneficial relationships with the host, the gut microbiome has the capacity to influence energy metabolism. While the precise mechanisms are not yet fully understood, there is a belief that the gut microbiome is linked to metabolic disorders in both humans and animals (69).

The connection between the gut microbiome and the consumption of NNS has been observed as far back as the early 1980s (69). During that period, it was discovered that exposure to saccharin could shift the balance between different types of bacteria in the gut. Male rats that consumed saccharin for a period of 10 days experienced a reduction in anaerobic gut bacteria and an increase in aerobic bacteria (70). This finding illustrated that specific microbiota in the gut could be influenced by dietary interventions. Furthermore, this evidence suggests that the adverse health effects linked to certain gut microbiota might be a consequence of NNS consumption. These studies underscore the notion that consuming NNS can disrupt gut microbiota, potentially promoting obesity and insulin resistance.

In a study by Ahmad et al. (59) the researchers sought to understand the influence of sucralose and aspartame intake on the gut microbiota using practical doses of NNS. This double-blind, randomized crossover trial engaged 17 healthy participants aged between 18 and 45, all with a BMI between 20 and 25. The findings revealed that in these healthy subjects, the ingestion of aspartame and sucralose didn't lead to notable changes in the composition of the gut microbiota (71). In research by Méndez-García et al. (72) it was found that daily intake of 48 mg of sucralose over a span of ten weeks led to an imbalance in the gut microbiome. This was evident by an increase in *Blautia coccoides* and a decrease in *Lactobacillus acidophilus* among healthy young adults who were not insulin-resistant (72). In a separate research conducted by Suez et al., it was demonstrated that three prevalent non-sugar sweeteners, namely saccharin, sucralose, and aspartame, influenced an elevation in glucose levels by altering the makeup of the gut microbiota (73).

Suez et al. (73) have recently proposed that NNS, including saccharin, sucralose, and aspartame, contribute to the development of glucose intolerance by causing changes in the composition and function of the intestinal microbiota. This research, has garnered significant attention from both the media and healthcare professionals (74).

Conflicting findings prompted researchers at the Weizmann Institute of Science in Israel to delve deeper into the impact of NNS on both mice and humans (75). They conducted a series of experiments initially on mice and later on humans. Their findings suggest that NNS consumption disrupts the composition of intestinal microbiota in mice, which, in turn, could potentially lead to metabolic imbalances like glucose intolerance. Furthermore, prior research has proposed that the contemporary “Western” diet, characterized by its high fat and high sugar content, has triggered alterations in the genetic makeup and metabolism of the human gut microbiota (76). It has been hypothesized that these changes may be contributing to the rising incidence of chronic illnesses in developed countries (75, 76).

## 5 Future perspective

The future of research on ASs must adopt a multifaceted approach to unravel their complex impacts on human health, particularly focusing on individuals with high consumption rates such as those with T2DM and Metabolic Syndrome. Critical areas for exploration include the long-term effects on metabolic health, the comprehensive impact on the gut microbiota across various populations including healthy individuals, those with metabolic disorders, and across different age groups from children to the elderly. Special attention should be given to understanding the systemic effects, including potential links to aging processes such as telomere length attrition, and the specific impacts on beneficial microbiota and metabolic pathways. Future studies should leverage advances in genomics, metabolomics, and microbiology to provide a deeper understanding of these interactions, aiming to inform more nuanced and evidence-based dietary recommendations. This research is essential for developing targeted interventions that mitigate the risks associated with artificial sweetener consumption, ultimately contributing to improved public health outcomes.

## 6 Conclusion

This review elucidates that ASs have significant benefits for diabetes care, particularly in terms of weight control and blood glucose level stabilization. Their connection with gut health, cellular aging, and insulin sensitivity, is still controversial. The extant literature has a dichotomy of findings, with some indicating potential health advantages and others indicating caution. While ASs are useful according to several scientific reports, they should be used with caution until more definite research into their broader health effects is available. Future research is critical for navigating the complex environment of ASs health effects that will ultimately give a clearer guidelines for individuals and healthcare professionals involved in diabetes management.

## Author contributions

MA: Conceptualization, Methodology, Writing – original draft, Writing – review & editing. JK: Conceptualization, Supervision, Validation, Writing – review & editing. LV: Formal analysis, Visualization, Writing – review & editing. AS: Writing – review & editing. VC: Writing – review & editing. RM: Writing – review & editing.
